# Contamination by respiratory viruses on outer surface of medical masks used by hospital healthcare workers

**DOI:** 10.1186/s12879-019-4109-x

**Published:** 2019-06-03

**Authors:** Abrar Ahmad Chughtai, Sacha Stelzer-Braid, William Rawlinson, Giulietta Pontivivo, Quanyi Wang, Yang Pan, Daitao Zhang, Yi Zhang, Lili Li, C. Raina MacIntyre

**Affiliations:** 1 School of Public Health and Community Medicine, UNSW Medicine, University of New South Wales, Level 2, Samuels Building, Sydney, 2052 Australia; 2 University of New South Wales, Virology Research Laboratory, Prince of Wales Hospital, Randwick, NSW 2031 Australia; 3 SAViD (Serology & Virology Division), Prince of Wales Hospital, Randwick, Australia; 4 Infection Prevention Management and Staff Health Services- St Vincent’s Hospital, Sydney, Australia; 5 Beijing Center for Diseases Prevention and Control, Beijing, China; 6 Fangshan Center for Diseases Prevention and Control, Beijing, China; 7 Biosecurity Program, The Kirby Institute, University of New South Wales, Sydney, NSW 2052 Australia; 8 College of Public Service & Community Solutions, and College of Health Solutions, Arizona State University, Phoenix, AZ 85004 USA

**Keywords:** Mask, Health care workers, Viruses, Infection control

## Abstract

**Background:**

Medical masks are commonly used in health care settings to protect healthcare workers (HCWs) from respiratory and other infections. Airborne respiratory pathogens may settle on the surface of used masks layers, resulting in contamination. The main aim of this study was to study the presence of viruses on the surface of medical masks.

**Methods:**

Two pilot studies in laboratory and clinical settings were carried out to determine the areas of masks likely to contain maximum viral particles. A laboratory study using a mannequin and fluorescent spray showed maximum particles concentrated on upper right, middle and left sections of the medical masks. These findings were confirmed through a small clinical study. The main study was then conducted in high-risk wards of three selected hospitals in Beijing China. Participants (*n* = 148) were asked to wear medical masks for a shift (6–8 h) or as long as they could tolerate. Used samples of medical masks were tested for presence of respiratory viruses in upper sections of the medical masks, in line with the pilot studies.

**Results:**

Overall virus positivity rate was 10.1% (15/148). Commonly isolated viruses from masks samples were *adenovirus* (*n* = 7), *bocavirus* (*n* = 2), *respiratory syncytial virus* (n = 2) and *influenza virus* (n = 2). Virus positivity was significantly higher in masks samples worn for > 6 h (14.1%, 14/99 versus 1.2%, 1/49, OR 7.9, 95% CI 1.01–61.99) and in samples used by participants who examined > 25 patients per day (16.9%, 12/71 versus 3.9%, 3/77, OR 5.02, 95% CI 1.35–18.60). Most of the participants (83.8%, 124/148) reported at least one problem associated with mask use. Commonly reported problems were pressure on face (16.9%, 25/148), breathing difficulty (12.2%, 18/148), discomfort (9.5% 14/148), trouble communicating with the patient (7.4%, 11/148) and headache (6.1%, 9/148).

**Conclusion:**

Respiratory pathogens on the outer surface of the used medical masks may result in self-contamination. The risk is higher with longer duration of mask use (> 6 h) and with higher rates of clinical contact. Protocols on duration of mask use should specify a maximum time of continuous use, and should consider guidance in high contact settings. Viruses were isolated from the upper sections of around 10% samples, but other sections of masks may also be contaminated. HCWs should be aware of these risks in order to protect themselves and people around them.

## Background

Infectious diseases are a continuing threat, with constant emergence or re-emergence of serious diseases in various parts of the world and healthcare workers (HCWs) are particularly at-risk of exposure to index cases [1–4]. Various types of personal protective equipment (PPE) are recommended and used by HCWs to protect from infections, including medical masks, respirators, gloves, gowns, goggles and face shield [5, 6]. In healthcare settings, medical masks are used by HCWs to protect from splashes and sprays of blood and body fluids, and by sick individuals to prevent spread of respiratory infections to others [7]. Reuse and extended use of masks are also common in many parts of the world, particularly during outbreaks and pandemics [8, 9]. Respiratory pathogens may be present on used masks layers and lead to infection of the wearer [10]. In hospital settings, these pathogens may be generated from breathing, coughing or sneezing patients or during aerosol generating medical procedures [11]. Studies have shown that influenza virus can remain airborne for 3 h after a patient has passed through an emergency department [12]. While using masks, or during long periods of time of re-using them, these pathogens may cause infection through hand or skin contamination, ingestion, or mucus membrane contact [10].

Currently there are limited data on the presence of respiratory pathogens on surface of PPE and other fomites in hospital settings. Previous studies show that influenza and respiratory syncytial virus (RSV) may survive on outer surface of PPE [11–14]. A study showed that influenza viruses may survive on hard surfaces for 24–48 h, on cloth up to 8–12 h and on hands for up to 5 min [13]. A previous study in an Australian Neonatal Intensive Care Unit (NICU), respiratory syncytial virus (RSV) RNA was identified from 4% of dress samples and 9% of environmental samples [14]. If health departments do not provide clear guidance on the use of masks in these situations, HCWs may continue using contaminated masks and may get infection [15]. The risk of self-contamination of HCWs is influenced by the mask itself, its shape and properties, and the virus concentration on its surface. To our knowledge, only one study examined the presence of contamination on mask and various bacteria were isolated from outer surface of medical masks [16]. The main aim of this study was to study the level of contamination on the surface of medical masks.

## Methods

### Pilot studies

Medical masks were tested as per protocols developed through two pilot studies in Sydney Australia.

#### Pilot study 1 (laboratory testing)

**Fig. 1:**
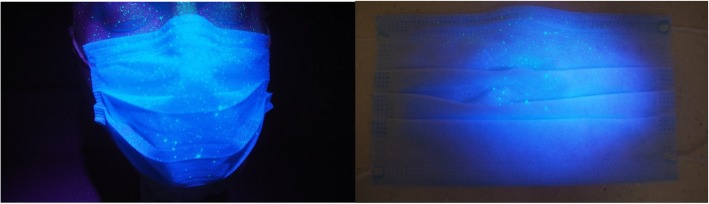
Fluorescent particles (UV Glow power) following spraying from 1 m from the front of the mask

**Fig. 2:**
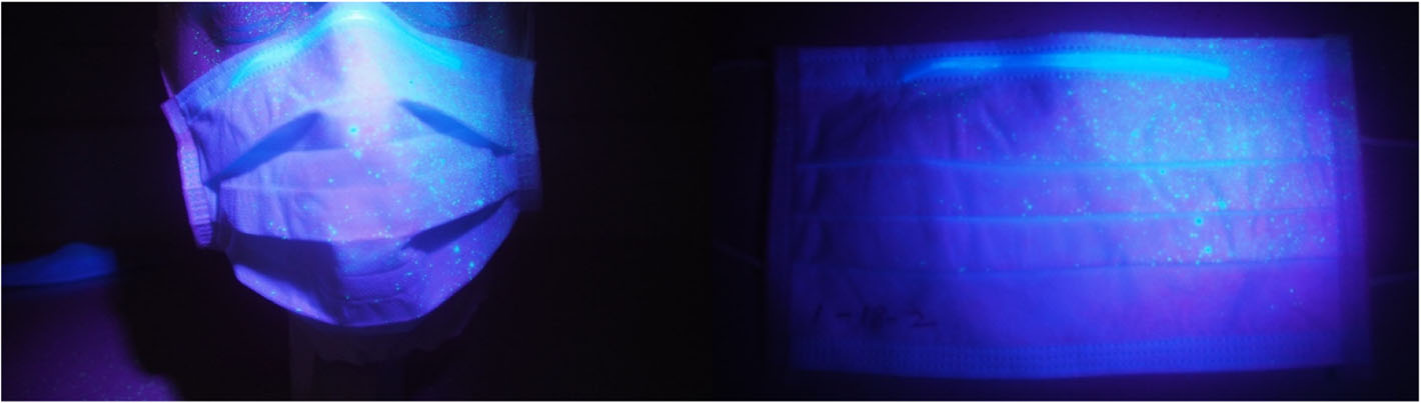
Fluorescent particles (UV Glow powder) following spraying from 1 m from the side of the mask

The aim of this pilot study was to identify areas of maximum virus concentration on the surface of masks. Medical masks were donned on a simple mannequin in a laboratory setting and fluorescent particles (UV Glow powder) were sprayed front on and side on from a distance of approximately 1 m using a spray bottle. We performed three experiments from the front and three experiments from the sides of mannequin. UV light was used to quantify the density of particles on mask surface and to identify area of maximum concentration. In all three experiments, most particles were concentrated on upper right, middle and left sections of the masks (Figs. 1 and 2).

#### Pilot study 2 (clinical testing)

The second pilot study was conducted in two tertiary referral hospitals in Sydney Australia to develop testing methodology. Twelve HCWs (doctors and nurses) from the infectious diseases, respiratory/ chest wards and intensive care unit (ICU) participated in the study. HCWs were asked to wear medical masks for a shift (minimum 30 min) used masks were tested in the Virology Research Laboratory, University of New South Wales and Prince of Wales Hospital Sydney Australia. If a respirator was indicated due to airborne inflictions, HCWs were excluded from the study and were allowed to use a respirator.

**Fig. 3:**
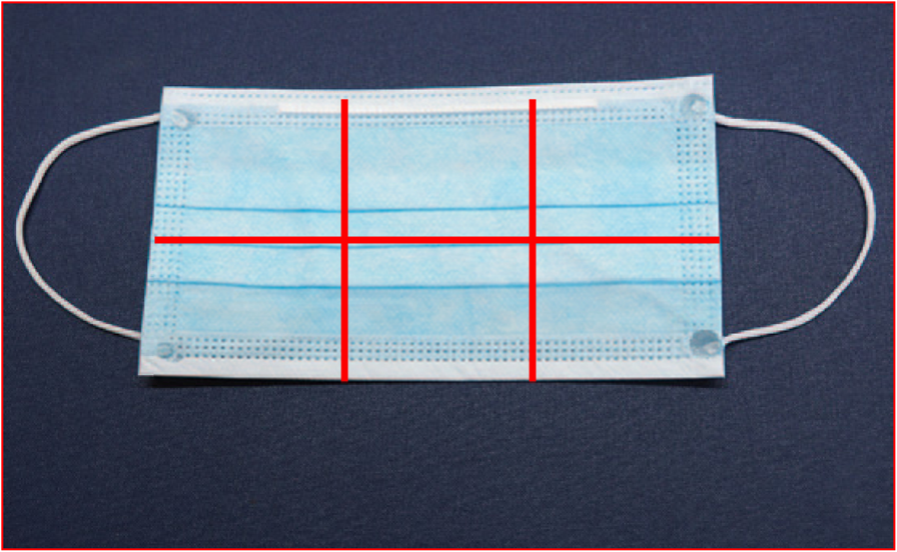
Sections of medical masks for testing

Medical masks were divided into six sections as shown in Fig. 3. Samples were taken from upper three sections of masks i.e. 36 samples were tested in total (12 masks X 3 samples). The outer layer of the mask was removed using sterile tweezers. The mask layer was placed into a 15 ml falcon tube containing 700 μl of Phosphate buffered saline and vortexed for 20 s. After 10 min incubation the mask was placed in a custom made filter tube inside an eppendorf tube and centrifuged briefly. The filtrate was then transferred to 1.5 ml Eppendorf tube. Total nucleic acid was extracted on the Kingfisher Flex (Thermo Scientific) using the MagNA Pure Total Nucleic Acid Isolation Kit (Roche) according to the manufacturer’s instructions. Presence of respiratory viruses was detected using the Seegene Allplex™ Respiratory Panel Assays 1,2,3 (Seegene).

### Main study

The main study was conducted in respiratory wards and fever clinics of three selected hospitals in Beijing China from December 2017 to January 2018. Doctors and nurses from selected wards were invited to participate in the study. Participants include nursing and medical staff aged > 18 years working full time in the ward who were able to provide written and informed consent. Participants with pre-existing respiratory, medical illness or pregnancy were excluded. As we did not test the participants, detail history on respiratory symptoms was taken to rule out contamination of masks by participants themselves.

HCWs from the participating wards were asked to wear medical masks for a shift (6–8 h), or as long as they could tolerate the masks with no adverse event. Three layered standard medical masks were used. If HCWs used more than one mask during their shift, first sample was collected and tested. Used medical masks were collected at the end of the day and were stored immediately in zip-lock bags. HCWs were advised to store masks in in zip-lock bags while they take off the masks during break time. All masks samples were labelled with participants’ ID and hospital ID. At the end of the study, HCWs were asked to complete a short survey to collect information on mask use in routine (type of mask used, number of masks used and situations when masks were normally used) and during the study period (wearing time, number of patients seen, situations when masks were used, aerosol generating procedures performed and hand hygiene during donning and doffing). Participants reported “number of masks used” and “number of patients seen” in absolute numbers. “Duration of mask use” was recorded in hours as, < 1 h, 1 to 2 h, 2 to 4 h, 5 to 6 h, 7 to 8 h, > 8 h. “Situations when masks were used” were categorized into: “used continuously”, “used continuously except during breaks”, “used only during patients’ encounters” and “used only high-risk patient encounters”.

### Mask testing for the main study

Medical masks were tested in the Beijing CDC laboratory. All masks were collected immediately after use in zip-lock bags and kept at − 80 °C until testing. Pilot studies showed that upper sections of masks were more contaminated (Figs. 1 and 2). The outer layers of upper right, middle and left mask were separated with a same size, placed into separated tubes containing 700 μl PBS buffer (Gibco, USA), vortexed for 1 min, and finally aliquoted 50 μl for viral testing. We performed three tests on upper right, middle and upper left sections of the masks on around a quarter mask sample (26%) and performed one test on the remaining mask samples (74%). For one testing, outer layers of upper right, middle and left section of mask were separated and placed into the same tube. Viral DNA/RNA was extracted using KingFisher Flex 96 viral DNA/RNA purification kit (Thermo Fisher, USA) according to the manufacturer’s instructions. The reverse-transcription polymerase chain reaction was performed to amplify 15 viral target genes, including influenza A/B virus, influenza A(H1N1) and A(H3N2), parainfluenza viruses 1–4, rhinoviruses, bocavirus, human metapneumovirus, adenovirus, respiratory syncytial virus, coronaviruses OC43, 229E, NL63 and HKU1 using a commercial multiplex combined real-time PCR detection kit for Respiratory virus, which is developed by “Jiangsu Uninovo Biological Technology Co. Ltd.” in China.

### Sample size

Currently there is very limited data on testing of masks surface for presence of pathogens. In previous studies influenza virus was detected on over 50% of the fomites tested in community settings during influenza season [17]. The rate is expected to be higher in the healthcare setting and moreover other viruses will also be tested. Assuming 25% higher positivity rate in the healthcare setting, the required sample size would be 134 masks, with 80% power and two-sided 5% significance level for detecting a significant difference. Some HCWs might not be able to provide mask samples, we aimed to recruited 145 HCWs in total for this study.

### Analysis

Descriptive analysis was conducted, and rates and frequencies were calculated. Univariate analysis was performed to identify the factors associated with mask positivity. Logistic regression was used to calculate odds ratio (OR) and 95% confidence intervals (CI) Data were analyzed in SAS (SAS Institute Inc., USA) version 9.4.

### Ethics and consent to participate

Ethics approval for pilot study was sought from South Eastern Sydney Local Health District (SESLHD). Ethics approval for the main study was sought from Human Research Ethics Committee UNSW (HC16703) and IBR China. Written consent as obtained from all participants.

## Results

Of 36 samples in pilot testing, three samples were positive for *human enterovirus*. Two samples were positive from outer sections of mask, while one sample was positive from middle section. No other viruses were detected in mask samples.

A total of 158 participants were recruited from three hospitals in the main study. Ten participants provided more than one samples for the testing, so we excluded these cases from analysis due to uncertainty around the duration of mask use being tested. Most participants were recruited from Hospital A (52%, 77/148), largely from the respiratory ward 47.3%, 70/148). Around half of the participants were doctors (45.9%, 68/148), and majority were female (81.8%, 121/148). In routine clinical practice, almost all participants (98.6%, 146/148) had previously used disposable medical masks. Generally, most of the participants had been using 1 or 2 medical masks per day (90.6%, 134/148) and around two third participant (68.2%, 101/148) had been using mask all the time during the clinical work (Table 1).

**Table 1:** Demographic data

Variables	Number (*n* = 148)	Percent
Hospital		
Hospital A	77	52.0
Hospital B	26	17.6
Hospital C	45	30.4
Ward		
Internal medicine	52	35.1
Respiratory	70	47.3
Pediatrics	26	17.6
Position		
Doctor	68	45.9
Nurse	80	54.1
Age		
≤ 30 year	41	27.7
31–40 years	68	45.9
≥ 41 years	39	26.4
Gender		
Male	27	18.2
Female	121	81.8
Type of mask normally used in the hospital		
Cloth re-usable facial masks	2	1.4
Disposable medical masks	146	98.6
Number of masks routinely used in the hospital		
1	46	31.1
2	88	59.5
3	10	6.8
4	4	2.7
When masks are normally used		
All the time	101	68.2
When treating certain patients	47	32.8

During the study period, around 2/3 participants used masks for > 6 h – “7–8 h” 80 participants (54.1%) and “> 8 h” 19 participants (12.8%). The remaining 1/3 used masks for ≤6 h – “1–2 h” 1 participant (0.7%), “3–4 h” 8 participants (5.4%) and “5–6 h” 40 participants (27%). Most participants (78.4%, 116/148) used masks either continuously or continuously except breaks. The majority of participants (83.8%,124/148) reported at least one problem associated with masks use. Commonly reported problems were pressure on face (16.9%, 25/148), breathing difficulty (12.2%, 18/148), discomfort (9.5% 14/148), trouble communicating with the patient (7.4%, 11/148) and headache (6.1%, 9/148). Majority of participants washed their hand during donning (91.2%, 135/148) /doffing (88.5%, 131/148) of medical masks and before (74.3%, 110/148) /after (85.1%, 126/148) touching patients. During the study period, 68% (101/148) participants used other PPE as well – mostly gloves and hair covers.

Overall virus positivity rate was 10.1% (15/148) and rates were similar after 1 testing on mask (10%, 11/110) compared to three testing (10.5%, 4 /38) (OR 1.06, 95% CI 0.32–3.55). *Adenovirus* was most commonly isolated from the masks (*n* = 7), followed by *bocavirus* (*n* = 2), RSV (n = 2) and *influenza* virus (n = 2) (Table 2).

**Table 2:** Pathogens isolated from outer surface of masks

Viruses	Positive in one test (Total tests 110)	Positive in three tests & sample location (Total test 38)
Adenovirus^a^	6	1 middle section of mask
Bocavirus^a^	2	0
Human metapneumovirus^a^	0	1 right section of mask
Influenza B & type 4 parainfluenza virus^b^	1	0
Influenza H1N1 & influenza B^c^	1	0
Respiratory syncytial virus^a^	1	1 middle section of mask
Type 2 parainfluenza virus^a^	0	1 right section of mask
Total positive (Positivity rate)	11 (9.4%)	4 (9.8%)

^a^ Isolated from internal medicine ward, ^b^ isolated from pediatric ward ^c^ isolated from respiratory ward

Compared to the participants working in internal medicine department, virus positivity rates were lower among those working in respiratory (OR 0.04, 95% CI 0.01–0.34) and pediatric (OR 0.12, 95% CI 0.01–0.97) departments. Virus positivity was significantly higher on masks samples worn by participants who used masks for > 6 h, compared to those who used mask for ≤6 h that day (OR 7.9, 95% CI 1.01–61.99). Similarly, virus positivity was significantly higher on masks samples worn by participants who examined > 25 patients per day, compared to who examined ≤25 patients (OR 5.02, 95% CI 1.35–18.60). Virus positivity rates were also higher in mask samples collected from males, participants who used mask during encounters with high risk patients and those who performed aerosol generating procedures (AGPs), however the difference was not statistically significant (Table 3).

**Table 3:** Factors associated with virus positivity on masks surface

Variables	Positive for any virus		Odds ratio (OR) (95% CI)
	Number	Percent	
Hospital			
Hospital A	12/77	15.6	Ref^a^
Hospital B	1/26	3.8	0.22 (0.03–1.75)
Hospital C	2/45	4.4	0.25 (0.05–1.18)
Ward			
Internal medicine department	13/52	25	Ref
Respiratory department	1/70	1.4	0.04 (0.01–0.34)^d^
Pediatrics department	1/26	3.8	0.12 (0.01–0.97)^d^
Gender			
Male	4/27	14.8	Ref
Female	11/121	9.1	0.57 (0.16–1.97)
Position			
Doctor	7/68	10.3	Ref
Nurse	8/80	10	0.97 (0.33–2.82)
Age			
≤ 30 years	5/41	12.2	Ref
31–40 years	5/68	7.4	0.57 (0.15–2.11)
≥ 41 years	5/39	12.8	1.06 (0.28–3.98)
Mask use time during the study			
≤ 6 h	1/49	2	Ref
> 6 h	14/99	14.1	7.9 (1.01–61.99)^d^
Patients’ seen			
≤ 25 cases	3/77	3.9	Ref
> 25 cases	12/71	16.9	5.02 (1.35–18.60)^d^
How medical masks were used			
Used continuously	4/28	14.3	Ref
Used continuously except breaks^b^	9/88	10.2	0.65 (0.19–2.22)
Used only during patients encounters	0/26	0	0.10 (0.01–2.12)
Used only high-risk patient encounters	2/6	33.3	3.02 (0.43–21.44)
Preformed AGPs ^c^ during the study			
No	7/95	7.4	Ref
Yes	8/53	15.1	2.24 (0.76–6.55)
Hand wash			
No	2/13	15.4	Ref
Yes	13/135	9.6	0.59 (0.12–2.94)

^a^ Reference ^b^ lunch, tea and toilet ^c^ aerosol generating procedures ^d^Significant results

## Discussion

To our knowledge this is the first study examining the presence of respiratory viruses on the outer surface of used medical masks. One in ten masks were positive for any virus which highlights the risk of self-contamination to the wearer, particularly on doffing [18]. Reuse and extended use of masks are very common, particularly in low income countries and during outbreaks and pandemics when supplies are short, and demand is high [19, 20]. Staff should be aware of the risk associated with the reuse and extended use of masks and respiratory protective devices and high clinical contact. Large scale studies should be conducted to determine the contamination on other PPEs as well and to quantify the risk of infection among HCWs.

Epidemics of a new infectious disease may be devastating due to global spread, disease burden and high case fatality. PPE are generally considered lowest among infection control hierarchy and recommended to be used with other administrative and environmental control measures [21]. However, masks, respirators and other PPE are important during initial phase of outbreak and pandemic when drugs and vaccine are not available [22]. PPE can easily get contaminated during clinical care of sick patients which may result in an increased risk of infection in wearer [18]. Many simulation studies have also shown presence of particles on the potential surface of PPE and associated risk of self-contamination during doffing of PPE [5, 22–24]. In this study we only tested the presence of viruses on the medical masks. Overall virus positivity rate in this was 10.1% (15/148) and adenovirus was isolated from 7 mask samples while bocavirus, RSV and influenza viruses were isolated from 2 samples each. Prospero et al. conducted a study in dental settings and estimated the bacterial contamination on surface of masks used by dentist, lamps, areas near spittoons, and mobile trays. Sterile nitrocellulose filters were applied on these surfaces to isolate pathogens. Highest levels of bacterial contamination (Streptococcus species 42%, Staphylococcus species 41%, and gram-negative bacteria 17%) were recorded on the external surface of masks wore by dentist [16]. Large scale studies should be conducted to examine presence of various pathogens on the surface of masks and other PPE.

In this study, the risk of mask contamination was associated with duration of masks use and number of patients seen. Currently there is no standard duration for the time period that facemasks and respirators can safely be used. Theoretically, there may be a risk of infection in wearer if contaminated masks are used for prolonged time. Currently there are no data around risk associated with reuse and extended used of masks and other PPE. One study showed that influenza virus may survive on mask surface and maintained infectivity for at least 8 h [25]. Our study showed very low infection among HCWs who used masks for ≤6 h. High virus positivity on masks samples worn by HCWs who examined > 25 patients, may be due to more frequent clinical contact with infective cases and transfer of more pathogens from patients to mask surface. Virus positivity rates were also higher in those working in internal medicine department compared to respiratory and pediatric departments. The reason of high virus positivity in internal medicine department is not clear, but this may be due to using varying infection control policies and practices. High risk perception and more infection control measures may result in low virus positivity in in respiratory and pediatric departments. However, the sample sizes and number of positive results were too low to make meaningful comparisons between departments. There is a need for more research to define the exact threshold of safe duration, and to develop a comprehensive policy on the use of masks in hospital settings and protocols should specify a maximum time of continuous use and should consider guidance in high contact settings.

We also aimed to identify the area on the mask surface with maximum respiratory virus concentration. Laboratory based pilot study showed maximum fluorescent contamination on upper sections of the masks, which is also the likely area to be touched on removal. Of the three positive tests in hospital-based pilot study, two samples were positive from outer sections of mask, while one sample was positive from middle section. In the main study we were able to check the location of contamination on a quarter of mask samples. Of the 38 mask samples, one or more viruses were isolated from four (10.5%) samples – two from middle section of masks and two from right section of the masks. This presents a large area of potential contamination which place HCW at risk when removing a mask. These data may assist in developing policies on for doffing of masks after encounter with infective cases. As a general rule, HCWs should not reuse masks, should restrict use to less than 6 h and avoid touching the outer surface of mask during doffing, and practice hand hygiene after removal.

There are limitations of this study. Due to funding constraints we tested selected masks samples. We performed three tests on a sub-sample (26%) to identify the area of maximum concentration. Moreover, we just tested upper three sections of medical masks based on the first pilot study, while lower three sections should also be tested. Then we tested only outer layer of masks and did not check filtering layer and inner layer due to funding constraints. Ideally all sections and layers of masks should be tested. We collected detail history from the participants to rule out any existing respiratory illness. Although none of the participant had a respiratory or a medical illness, it is not possible to determine whether viruses isolated from the masks surface were from exogenous or endogenous source. For example, adenovirus was most commonly identified in this study and is associated with mild or no respiratory illnesses. Ideally participants should also be swabbed to rule out infections, and the inside surface should also be tested. However, given the large variations of infection probability in different types of wards, it is unlikely that all viruses came from the background infection. To overcome this limitation, detailed history on respiratory symptoms was taken to rule out contamination of masks by clinically ill participants themselves. Moreover, we only examined viruses on the masks, while bacteria and other pathogens may also be present [16]. Mask use was not monitored, and self-reported compliance was recorded. Previous studies show that self-reported compliance is generally reported to be higher compared to the actual compliance [26, 27]. We also did not document the method of mask removal, nor the number of times the HCW touched the mask.

## Conclusion

To maintain the functionality and capacity of the health care workforce during outbreaks or pandemics of emerging infections, HCWs need to be protected. This study provides new data, which will help developing policies for safe workplace environment. The study shows that the prolonged use of medical masks (> 6 h) and frequent clinical contact in healthcare setting increase the risk to health workers through contaminated PPE. Protocols on duration of mask use should specify a maximum time of continuous use.

## Data Availability

The datasets used and/or analysed during the current study are available from the corresponding author on reasonable request.

## Abbreviations

AGPs: Aerosol generating procedures
HCW: Healthcare workers
ICU: Intensive care unit
NICU: Neonatal Intensive Care Unit
PPE: Personal protective equipment
RSV: Respiratory syncytial virus
SESLHD: South Sydney Local Health District
